# Glucocorticoid sparing effect of Janus kinase inhibitors compared to biologic disease modifying anti-rheumatic drugs in rheumatoid arthritis, a single-centre retrospective analysis

**DOI:** 10.1093/rheumatology/keae455

**Published:** 2024-08-21

**Authors:** Giovanni Adami, Riccardo Bixio, Giulia Virelli, Isotta Galvagni, Francesca Mastropaolo, Andrea Morciano, Francesca Ruzzon, Valeria Messina, Elena Fracassi, Davide Gatti, Ombretta Viapiana, Antonio Carletto, Maurizio Rossini

**Affiliations:** Rheumatology Unit, University of Verona, Verona, Italy; Rheumatology Unit, University of Verona, Verona, Italy; Rheumatology Unit, University of Verona, Verona, Italy; Rheumatology Unit, University of Verona, Verona, Italy; Rheumatology Unit, University of Verona, Verona, Italy; Rheumatology Unit, University of Verona, Verona, Italy; Rheumatology Unit, University of Verona, Verona, Italy; Rheumatology Unit, University of Verona, Verona, Italy; Rheumatology Unit, University of Verona, Verona, Italy; Rheumatology Unit, University of Verona, Verona, Italy; Rheumatology Unit, University of Verona, Verona, Italy; Rheumatology Unit, University of Verona, Verona, Italy; Rheumatology Unit, University of Verona, Verona, Italy

**Keywords:** TNFi, JAKi, glucocorticoid, baricitinib, tofacitinib, upadacitinib, filgotinib

## Abstract

**Background:**

Glucocorticoid sparing in rheumatoid arthritis (RA) treatment is crucial to minimizing adverse effects associated with long-term use. Janus kinase inhibitors (JAKi) could potentially offer a more potent glucocorticoid-sparing effect than biologic DMARDs (bDMARDs)

**Material and methods:**

This is a single-centre retrospective analysis of RA patients treated with JAKi or bDMARDs. Glucocorticoid tapering, rescue therapy and discontinuation were analysed through mixed-effects models, Poisson regression and multivariable logistic regression, respectively, adjusting for baseline disease activity, demographic factors and treatment line.

**Results:**

A total of 716 RA patients treated with JAKi (*n* = 156) or bDMARDs (*n* = 560) were evaluated. JAKi treatment was associated with a more rapid reduction in glucocorticoid dose within the first 6 months and 60% higher odds of discontinuation compared with bDMARDs (adjusted odds ratio 1.63; 95% CI: 1.02, 2.60, *P* = 0.039). Despite a higher baseline glucocorticoid dose, over 50% of JAKi-treated patients discontinued glucocorticoids after 12 months, *vs* ∼40% for bDMARDs. The need for glucocorticoid rescue therapy was significantly higher in the bDMARD group (rate ratio 2.66; 95% CI: 1.88, 3.74).

**Conclusion:**

Our findings indicate that JAKi facilitate more rapid glucocorticoid tapering compared with bDMARDs in RA patients. These results underscore the potential of JAKi to reduce long-term glucocorticoid exposure, highlighting their value in RA management strategies, including minimizing glucocorticoid-related adverse effects.

Rheumatology key messages

- Reducing and discontinuing glucocorticoids is important in rheumatoid arthritis.
- JAKi were associated with a more rapid tapering of glucocorticoids in rheumatoid arthritis patients.
- Discontinuation of glucocorticoids was more common in patients using JAKi and rescue therapy was less commonly used in patients treated with JAKi.

## Introduction

Rheumatoid arthritis (RA) is a systemic inflammatory disease characterized by erosive arthritis that can lead to joint deformities and severe disability [1]. Despite the availability of numerous DMARDs, glucocorticoids are widely prescribed for RA. However, their use is often continued chronically, irrespective of the bridging role advised by EULAR and ACR guidelines [2, 3]. The prolonged use of glucocorticoids could lead to relevant side effects such as increased risk of infections, cardiovascular events and osteoporosis [4–6]. Moreover, there is mounting evidence that even low-dose and short-term use of glucocorticoids might increase the rate of adverse events [7, 8]. Thus, minimizing glucocorticoids to the lowest effective dose is a key challenge in contemporary rheumatology, with rescue therapy frequently required in RA patients [9]. The glucocorticoid-sparing effect of biologic DMARDs (bDMARDs) and Janus kinase inhibitors (JAKi) remains under debate, although JAKi show promise in this context [10, 11]. In particular, a few observational studies showed that JAKi might permit a significant and rapid reduction of glucocorticoids even in patients with long-standing arthritis. However, such studies lacked a control group and the sample size was small [11, 12]. Randomized controlled trials often fall short in this context because glucocorticoid doses are usually maintained stable for the first 24 weeks, allowing little to no rescue therapy. Therefore, our study aims to investigate the glucocorticoid sparing effect of JAKi compared with bDMARDs.

## Methods

### Study design

This is a single-centre observational retrospective analysis of prospectively collected data of RA patients. Data were derived from the registry of the University of Verona (REUMABANK) which includes patients with inflammatory arthritis (including RA) from 2016 and followed prospectively and indefinitely. The University of Verona is a tertiary care centre with >1500 in-patient beds. The Section of Rheumatology dispose of eight in-patient beds and a large out-patient facility with seven consultants and >20 fellows. G.A., A.C., D.G. and O.V. were involved in primary data collection with the help of fellows for data entry.

### Study population

Clinical, demographic and laboratory data are collected at each visit that is scheduled as per normal practice every 3 months (±1 month). All patients with diagnosis of inflammatory arthritis that provide written informed consent are included in the registry. For the present analysis we included patients with a diagnosis of RA fulfilling the 2010 ACR/EULAR classification criteria (from January 2016 to January 2024), older than 18 years, with stable conventional synthetic DMARD therapy in the last 12 weeks, who were candidates to start either a JAKi or a bDMARD. The choice of treatment was at the discretion of the treating rheumatologist. Disease activity was assessed using disease activity score for 28 joints (DAS28) based on C reactive protein (CRP). Patients were seen as per clinical practice every 3 months (±1 month) and oral glucocorticoid dose was meticulously assessed at every visit. Patients were followed up to drug switch or discontinuation (as concern JAKi and bDMARDs) or last follow-up available. However, after switching, patients restarted to contribute to the analysis in the new group in which they switched. Missing variables were imputed with multiple imputation using logistic regression for categorical variables and linear regression with predictive mean matching for continuous variables. Missing data were <5% for every variable in the registry (Supplementary Materials, available at *Rheumatology* online).

### Statistical analyses

Descriptive statistics are provided. Student’s *t*-test, the Mann–Whitney *U*-test and the χ^2^ test were performed to assess group differences as appropriate. In the present study we focused on glucocorticoids tapering, discontinuation and rescue therapy in patients receiving JAKi *vs* bDMARDs. All differences were considered significant when the *P*-value was <0.05. All statistical analyses were performed using SPSS Statistics Version 26 (IBM Corp., Armonk, NY, USA) and GraphPad Prism version 10.2.2 (GraphPad Software, Boston, MA, USA). This study was approved by the University of Verona ethics committee (prot. REUMABANK 1483 CESC). All patients provided written informed consent to participate in the study.

### Disease activity

Differences in change from baseline in DAS28 were assessed using a mixed-effects model for repeated measures, which included: treatment, treatment by visit interaction, glucocorticoid dose at baseline, DAS28 at baseline, age, sex, presence of erosive arthritis, therapeutic line (first-line *vs* subsequent lines) and seropositivity status as fixed effects, and patients as the random effect. Least-squares (LS) mean, 95% CI and *P*-value were obtained from the model. Variables included in the model were selected based on the association in the univariate model and, if not significant, based on the opinion of the senior authors and literature review.

### Tapering of glucocorticoids

In this analysis we included only glucocorticoid users at bDMARD or JAKi initiation. Differences in change from baseline in glucocorticoid daily dose were assessed using a mixed-effects model for repeated measures, which included: treatment, treatment by visit interaction, glucocorticoid dose at baseline, DAS28 at baseline, age, sex, presence of erosive arthritis, therapeutic line (first-line *vs* subsequent lines) and seropositivity status as fixed effects, and patients as the random effect. LS mean, 95% CI and *P*-value were obtained from the model. Variables included in the model were selected based on the association in the univariate model and, if not significant, based on the opinion of the senior authors and literature review. Glucocorticoid dose was handled as a covariate in the mixed model and therefore a few patients might have discontinued and then restarted the drug within the 24-month period. These patients were kept in the analysis and dose was averaged.

### Glucocorticoid rescue therapy and flares

We employed a Poisson regression model to compare the rate ratios (RR) of glucocorticoid rescue therapy and flares between patients receiving JAKi and those treated with bDMARDs. The primary outcome variables were: counts of visits with glucocorticoid rescue therapy (defined as any increase in glucocorticoid dose from previous visit—both patients with and without baseline glucocorticoids were included in the analysis because they could increase the dose), the occurrence of moderate flares (defined as a ≥1.2 increase in DAS28 with prior DAS28 < 3.6) and the occurrence of minor flares (defined as a ≥ 0.6 increase in DAS28 with prior DAS28 < 3.6). Covariates included in the model were DAS28 at start of treatment course, glucocorticoid dose at start of treatment course, duration of follow-up, age, sex, presence of erosive arthritis, therapeutic line (first-line *vs* subsequent lines) and seropositivity status. The target variable was therapy type, stratified into two groups: patients on JAKi and those on bDMARDs. The 95% CI for the adjusted rate ratios were reported.

### Glucocorticoid discontinuation

In this analysis we included only glucocorticoid users at bDMARD or JAKi initiation. We conducted a multivariable logistic regression to evaluate the adjusted odds ratio (OR) of glucocorticoid discontinuation among patients receiving JAKi compared with those treated with bDMARDs, incorporating the same set of covariates as in the Poisson regression model to ensure consistency in adjustment for potential confounders (excluding duration of follow-up). Patients were followed until discontinuation and censored after discontinuation; therefore, patients restarting glucocorticoids within the first 24-month period of the analysis were not considered. We acknowledge that this might somehow bias the result since these patients might not be considered as ‘true’ discontinuers. For this reason, we have conducted a sensitivity analysis excluding early restarters.

## Results

Seven hundred and sixteen patients were evaluated. Descriptive characteristics of the cohort are reported in Table 1. In summary, bDMARD therapy was predominant, encompassing 78.2% (*n* = 560) of patients, with adalimumab being the most prescribed agent (*n* = 305, 54.5% of the bDMARD subgroup). Other frequently used bDMARDs included abatacept and etanercept (*n* = 87, 15.5% and *n* = 77, 13.7% of the bDMARD subgroup, respectively). Other treatments were less common, each constituting <1% of the bDMARD subgroup. JAKi accounted for 21.8% (*n* = 156) of patients, with baricitinib and upadacitinib being the preferred JAKi (*n* = 77, 49.36% and *n* = 60, 38.46% of the JAKi subgroup, respectively). Tofacitinib and filgotinib were less utilized (*n* = 18, 11.5% and *n* = 1, 0.6% of the JAKi subgroup, respectively).

**Table 1.** Baseline descriptive characteristics of the cohort

**Characteristic**	JAKi (*n* = 156)	bDMARD (*n* = 560)	*P*-value
Age, mean (s.d.), years	57.9 (11.9)	59.0 (13.1)	0.2912
Sex, female, *n* (%)	126 (81)	426 (76)	0.1767
Seropositive, *n* (%)	76 (49)	314 (56)	0.0871
Erosive RA, *n* (%)	62 (40)	319 (57)	<0.0001
Smoker, *n* (%)	17 (11)	67 (12)	0.9801
Treatment line, *n* (%)			
First	59 (38)	459 (82)	0.0010
≥Second	97 (62)	101 (18)	
Monotherapy, *n* (%)	95 (61)	319 (57)	0.8219
Follow-up, median (IQR), days	580 (670–505)	690 (950–415)	<0.0001
JAKi, *n* (%)			
Baricitinib	77 (49.4)		
Upadacitinib	60 (38.5)		
Tofacitinib	18 (11.5)		
Filgotinib	1 (0.6)		
bDMARD, *n* (%)			
Adalimumab		305 (54.5)	
Abatacept		87 (15.5)	
Etanercept		77 (13.8)	
Tocilizumab		46 (8.2)	
Certolizumab pegol		23 (4.1)	
Golimumab		14 (2.5)	
Infliximab		5 (0.9)	
Rituximab		2 (0.4)	
Sarilumab		1 (0.2)	
Taking glucocorticoids at baseline, *n* (%)	83 (53)	269 (48)	
Glucocorticoid dose at baseline, median (IQR), mg/day	3 (11–3.5)	2 (4–1)	0.0010
DAS28-CRP at baseline, mean (s.d.)	4.3 (1.1)	3.4 (1.2)	<0.0001

bDMARD: biologic DMARD; JAKi: Janus kinase inhibitor.

### Disease activity

**Figure 1.**
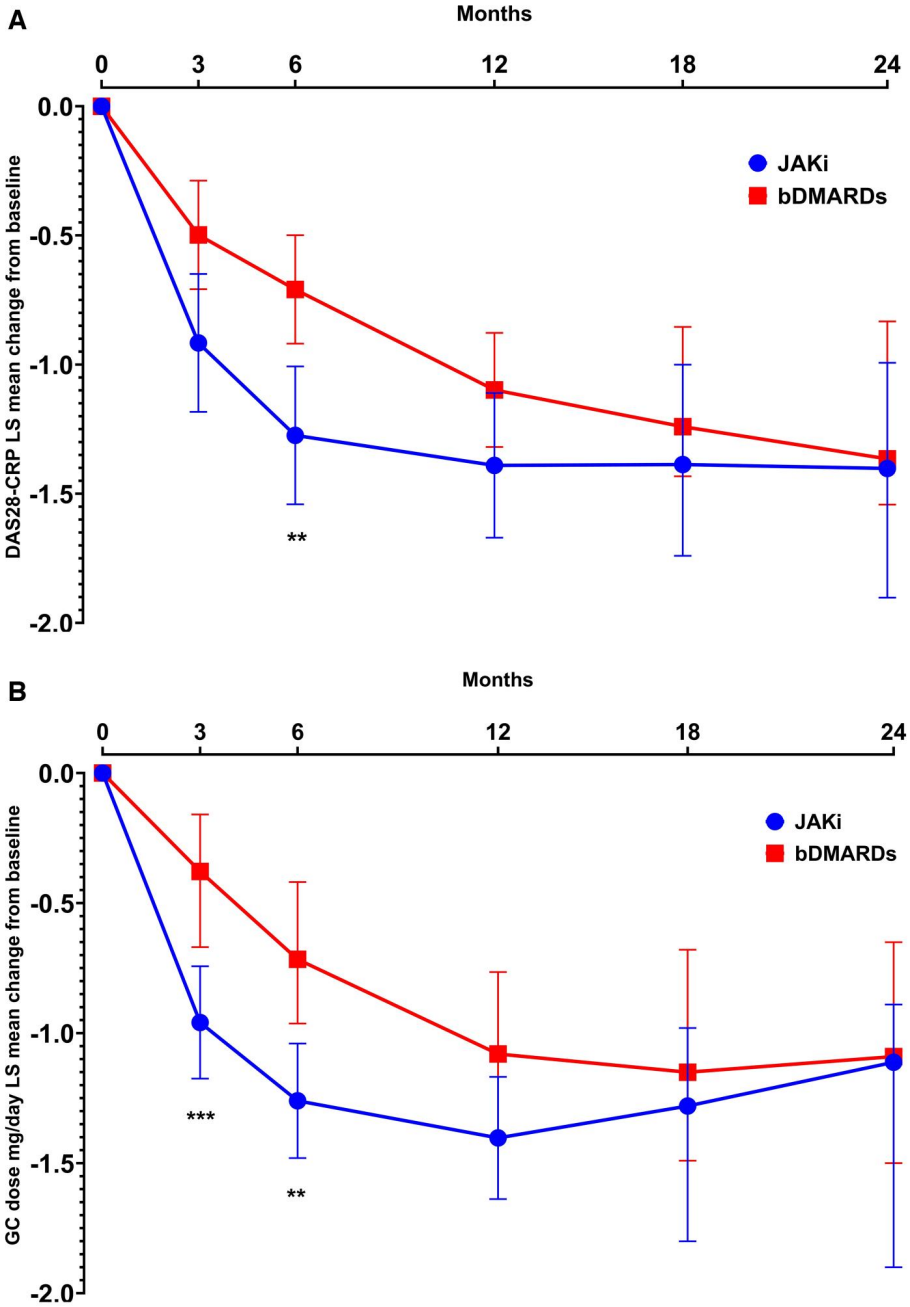
Least-squares mean change in DAS28-CRP (**A**) and glucocorticoid dose mg/day (**B**). bDMARDs: biologic DMARDs; DAS28: disease activity score for 28 joints; JAKi: Janus kinase inhibitors; LS: least squares

Improvements in DAS28 were seen as early as 3 months of follow-up in both groups. At 6 months DAS28 decrease was slightly greater with JAKi compared with bDMARDs (Fig. 1A).

### Tapering of glucocorticoids

Glucocorticoid dose decreased significantly in both groups but more rapidly in JAKi (Fig. 1B). LS mean change in glucocorticoid dose from baseline to month 3 was −0.95 mg/day (95% CI: −0.74, −1.17) in JAKi compared with −0.38 mg/day (95% CI: −0.16, −0.67) with bDMARDs, *P* < 0.0001. At 6 months LS mean change in glucocorticoid dose was −1.26 mg/day (95% CI: −1.48, −1.04) in JAKi compared with −0.71 mg/day (95% CI: −0.96, −0.42). After the 6-month time point differences were not significant.

### Glucocorticoid rescue therapy

**Figure 2.**
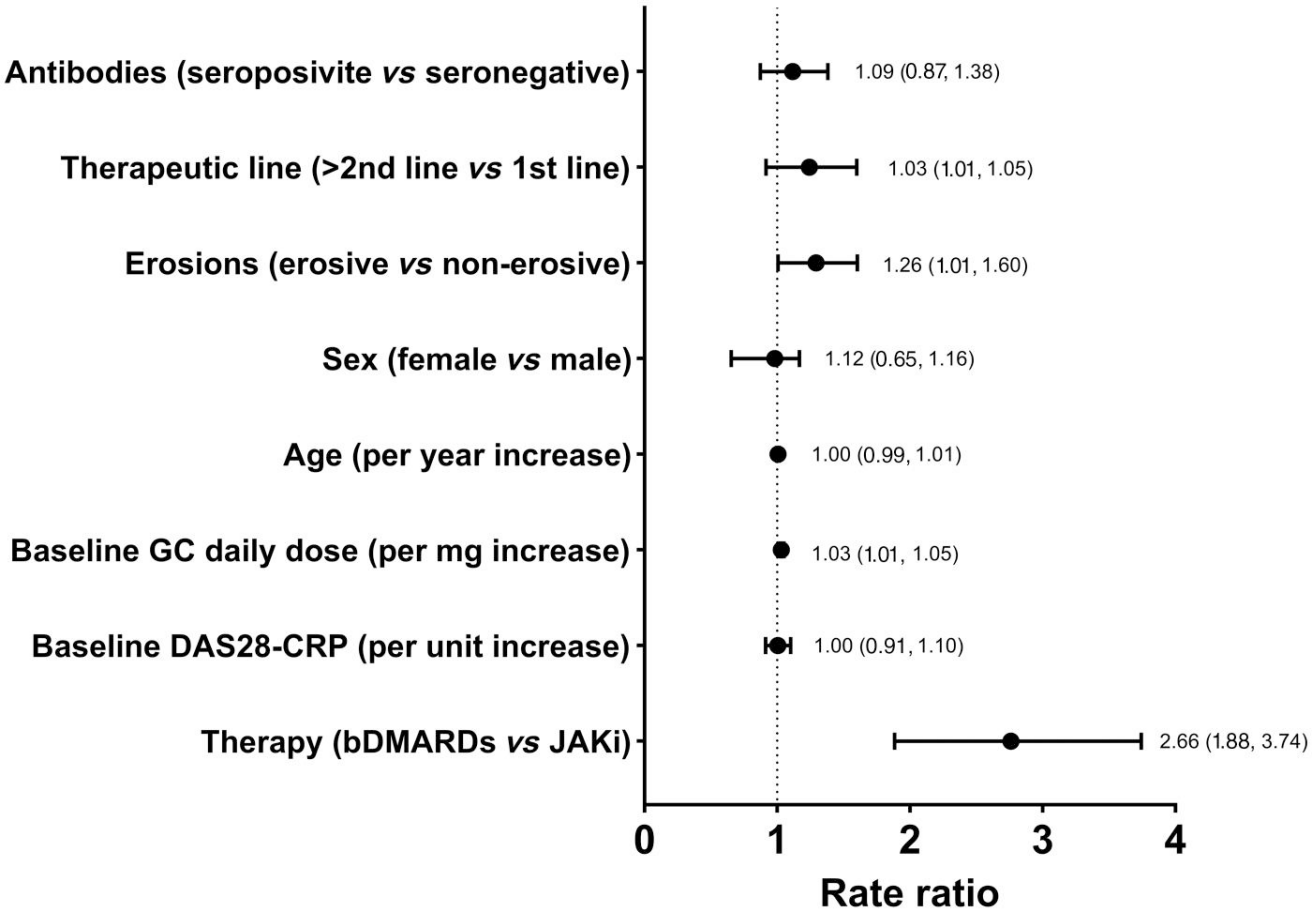
Rate ratio (RR) and 95% confidence intervals for the need of glucocorticoid rescue therapy over the follow-up period. RR indicates the factor by which the event rate increases (for RR >1) or decreases (for RR <1) for a 1 unit change in the predictor. For binary variables the RR represents how the event rate differs between the two categories. bDMARDs: biologic DMARDs; DAS28: disease activity score for 28 joints; JAKi: Janus kinase inhibitors

The need for glucocorticoid rescue therapy was more common in the bDMARDs group, showing a RR of 2.66 (95% CI: 1.88, 3.74). In Fig. 2 are shown the RR derived from the Poisson regression. Erosive RA and baseline glucocorticoid dose were predictors of the need for glucocorticoid rescue therapy over the follow-up. The occurrence of flares was more common in the bDMARDs group compared with JAKi with a RR of 2.13 (95% CI: 1.41, 3.20) for flares defined as DAS28 increase ≥1.2 points and 2.05 (95% CI: 1.51, 2.79) for flares defined as DAS28 increase ≥0.6 points.

### Glucocorticoid discontinuation

**Figure 3.**
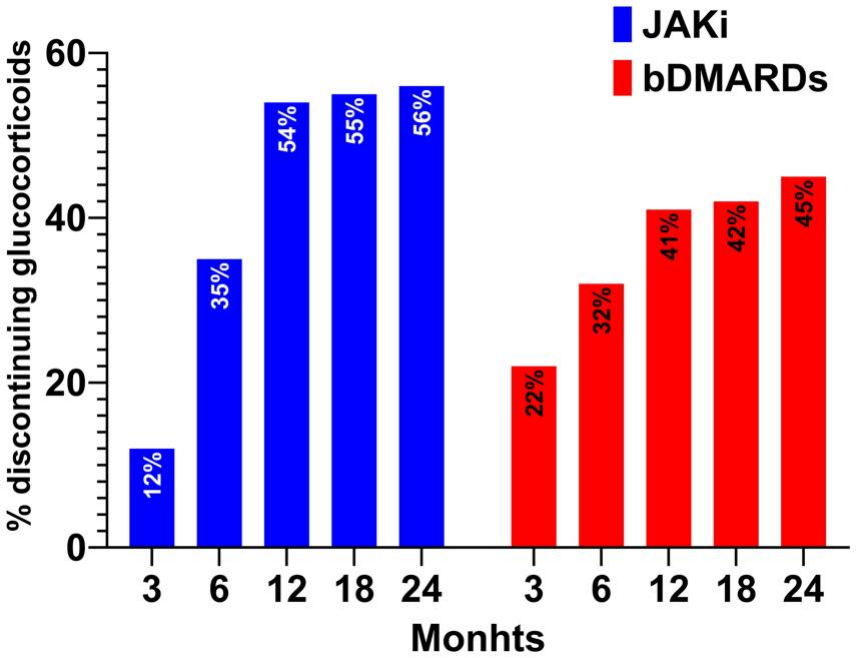
Cumulative proportion of patients discontinuing glucocorticoid among glucocorticoid users at baseline. bDMARDs: biologic DMARDs; JAKi: Janus kinase inhibitors

Among glucocorticoid users, 12%, 35%, 54%, 55% and 56% of patients starting a JAKi (*n* = 83) discontinued glucocorticoids at month 3, month 6, month 12, month 18 and month 24, respectively, compared with 22% 32%, 41%, 42% and 45% of patients starting a bDMARD (*n* = 269) (cumulative proportions of patients discontinuing glucocorticoids are reported in Fig. 3). Twenty-one patients (17 for bDMARDs and 4 JAKi) restarted the glucocorticoids after discontinuation within the 24-month period. In the multivariable logistic regression model, glucocorticoid dose at baseline was the strongest predictor for glucocorticoid discontinuation over the follow-up (adjusted OR [aOR] 0.93; 95% CI: 0.90, 0.97; *P* = 0.0006), and JAKi were associated with 60% greater chance of discontinuing glucocorticoids compared with bDMARDs (aOR 1.63; 95% CI: 1.02, 2.60; *P* = 0.039). The full results of the logistic regression are shown in Supplementary Table S1, available at *Rheumatology* online. In sensitivity analysis we excluded patients that restarted after discontinuation (*n* = 21) and the results of the model did not differ significantly (data not shown).

## Discussion

Our study aimed to compare the glucocorticoid-sparing effect of JAKi with bDMARDs. In aggregate we found that JAKi were associated with a more rapid glucocorticoid dose reduction that was paralleled by a reduction in disease activity. Glucocorticoid dose at baseline was the strongest predictor for glucocorticoid discontinuation over the follow-up but the odds for glucocorticoid definitive discontinuation were higher with JAKi compared with bDMARDs. This difference, however, seemed to vanish beyond 12 months of treatment. Indeed, the glucocorticoid-sparing effect of JAKi was proportionally higher at the beginning of the treatment, with the highest difference between JAKi and bDMARDs seen after 3–6 months. Yet, patients treated with JAKi used glucocorticoid rescue therapy less frequently compared with those treated with bDMARDs over the follow-up period. A possible, and logical, explanation could be the greater efficacy and effectiveness of JAKi in controlling pain in patients with RA compared with bDMARDs. A quick decrease in disease activity and pain scores with JAKi has been extensively described in observational and randomized clinical trials [13–16]. Thanks to their rapid effect on pain, JAKi could be able to reduce glucocorticoids sooner after their initiation, addressing the bridging role traditionally covered by glucocorticoids at treatment initiation, a period in which controlling pain and inflammation is more crucial than modifying the course of the disease. In our study we found that the crude proportion of patients discontinuing glucocorticoids within the first 3 months was higher for bDMARDs compared with JAKi (22% *vs* 12%, albeit not significant). The latter controversial finding seems to contradict the results of the dose tapering. However, patients on JAKi started at a higher dose of GCs on average, making a complete discontinuation more ambitious in such a short term. This can explain why we found the discrepancy between dose reduction (tapering) and crude analysis of discontinuation in the first 3 months. The glucocorticoid-sparing effect described in our study is indeed particularly relevant when considering that patients treated with JAKi had a higher baseline dose of glucocorticoids and were on average in more advanced lines of treatment, which could underlie a more active or more refractory disease. Nonetheless, to mitigate potential confounding effects, we adjusted for baseline disease activity, initial glucocorticoid dose and other relevant covariates in our mixed-effects model. This approach likely minimized the influence of these factors. Notably, over 50% of the JAKi-treated patients discontinued glucocorticoid use after 12 months, compared with ∼40% of those receiving bDMARDs. Albeit not significant, such a pattern supports the presence of a true glucocorticoid-sparing effect attributable to JAKi treatment. Indeed, a confounding effect due to higher baseline glucocorticoid doses would have resulted in similar discontinuation rates between the two groups at the 12-month mark or beyond, which was not the case. Finally, the proportion of patients on JAKi discontinuing at 12 months was somewhat in line with the one described by Spinelli and Guidelli in Italian RA patients treated with tofacitinib and baricitinib, respectively [11, 12]. Furthermore, the reduction in glucocorticoid daily dose was somewhat similar across these studies and in other uncontrolled populations of JAKi users. For example, Conigliaro *et al.* and Scheepers *et al.* reported that mean glucocorticoid dose decreased from ∼4.5 mg/day at JAKi initiation to 2.5 mg/day at follow-up (ranging from 12 to 24 months) with similar proportion of patients discontinuing completely the glucocorticoids [17, 18].

The observed trend towards more frequent glucocorticoid rescue therapy in patients treated with bDMARDs further substantiates our results and raises intriguing questions about the glucocorticoid-sparing capacity of these therapies. One hypothesis is that, while they effectively control systemic inflammation and joint damage over the long term, bDMARDs may not address acute flare-ups as rapidly or as effectively as JAKi, necessitating temporary increases in glucocorticoid dosage to manage breakthrough disease activity. This finding challenges the expectation that bDMARDs uniformly reduce the need for glucocorticoids across all aspects of RA management. It also suggests that certain subpopulations of RA patients or specific RA phenotypes may not achieve complete glucocorticoid discontinuation with bDMARD therapy. It has been shown consistently in the literature that patients treated with bDMARDs, particularly those receiving TNF inhibitors, may continue to see disease progression, including erosions, even while in remission [19–21]. Moreover, the occurrence of flare-ups is frequent among patients receiving bDMARDs. We previously showed that, with bDMARDs, the reduction of glucocorticoids below a certain threshold (around 2.5 mg/day) prompted an increase in flare rates [22]. Herein, we showed that the occurrence of flares was more common in the bDMARDs group compared with JAKi and the latter class of medications can minimize both the long-term and short-term use of glucocorticoids in RA, mitigating adverse effects and possibly improving patient outcomes. Interestingly, and in line with our findings, a real-world study published by Iwamoto and colleagues showed that the concomitant use of glucocorticoids did not enhance the efficacy of the JAKi in RA [23]. The authors speculated that, when using a JAKi, glucocorticoids might not be necessary as an adjuvant therapy. In aggregate, these results hold particular significance due to the established relationship between glucocorticoid therapy and an increased risk of adverse events, which is both time and dose dependent. It is well-established that prolonged and higher-dose glucocorticoid use in RA treatment is linked to a greater likelihood of fractures, cardiovascular events and infections. However, recent studies showed that even low-dose and short-term exposure to glucocorticoids have been associated with certain adverse outcomes, including fractures [7], cardiovascular events [6, 8] and infections [5]. By potentially reducing the need for glucocorticoids, JAKi offer a promising avenue to mitigate these risks. The ability to lower glucocorticoid doses or discontinue their use altogether without compromising disease control could significantly decrease the incidence of glucocorticoid-related adverse effects, thereby improving the overall safety profile of RA treatment regimens.

Our study has strengths and limitations. The main strength is the longitudinal design, allowing for dynamic observation of glucocorticoid tapering and discontinuation among RA patients. The inclusion of a comprehensive set of covariates in our analyses, such as baseline disease activity, glucocorticoid dose and demographic factors, strengthens the validity of our findings by minimizing potential confounding effects. Furthermore, the comparison between JAKi and bDMARDs provides valuable insights into the glucocorticoid-sparing effects of these treatments in a real-world setting. However, our study is not without limitations. The retrospective nature of the analysis may introduce selection bias, as treatment assignment was not randomized. Additionally, our single-centre design may limit the generalizability of the results to broader populations. The reliance on routinely collected clinical data also poses a risk of missing or inaccurate information, potentially affecting the precision of our findings. Moreover, we could not distinguish among different mechanisms of action across the spectrum of bDMARDs or across different JAKi.

In conclusion, our study demonstrates that JAKi offer a more rapid glucocorticoid dose reduction and higher odds of discontinuation compared with bDMARDs in RA patients, with significant differences observed primarily within the first 6 months of treatment. These findings suggest a potent glucocorticoid-sparing effect of JAK inhibitors, highlighting their potential role in minimizing long-term glucocorticoid use and associated adverse effects in RA management.

## Supplementary Material

keae455_Supplementary_Data

## Data Availability

The data underlying this article will be shared on reasonable request to the corresponding author.
