# Supplementary material for: Glucocorticoid sparing effect of Janus kinase inhibitors compared to biologic disease modifying anti-rheumatic drugs in rheumatoid arthritis, a single-centre retrospective analysis
Source: Rheumatology (Oxford). 2024 Aug 21;64(4):1698–704. doi: 10.1093/rheumatology/keae455 (PMC11962976; doi:10.1093/rheumatology/keae455)
Supplement: keae455_Supplementary_Data [file keae455_supplementary_data.docx]

**Supplementary materials**

**Glucocorticoid sparing effect of Janus Kinase inhibitors (JAKi) compared to biological Disease Modifying Anti-Rheumatic Drugs (bDMARDs) in rheumatoid arthritis: a single-center analysis**

**Authors:** Giovanni Adami^1^*, Riccardo Bixio^1^*, Giulia Virelli^1^, Isotta Galvagni^1^, Francesca Mastropaolo^1^, Andrea Morciano^1^, Francesca Ruzzon^1^, Valeria Messina^1^, Elena Fracassi^1^, Davide Gatti^1^, Ombretta Viapiana^1^, Antonio Carletto^1^^**^, Maurizio Rossini^1**^

1. Rheumatology Unit, University of Verona, Verona, Italy

*Dr. Adami and Dr. Bixio are dual first authors and contributed equally to this work

**Prof. Rossini and Dr. Carletto are co-senior authors and contributed equally to this work

**Table of content**

**Table s1.** Predictors of glucocorticoid discontinuation over the follow-up period

**Table s2.** Missing variables in the dataset

**Table s1.** Predictors of glucocorticoid discontinuation over the follow-up period

| **Covariates** | **aOR** | **95% CI** | **p value** |
| --- | --- | --- | --- |
| **Therapy (JAKi vs bDMARDs)** | **1.63** | **1.02 to 2.56** | **0.0392** |
| **DAS28-CRP baseline** | 1.051 | 0.89 to 1.24 | 0.5589 |
| **Glucocorticoid dose baseline mg/day of prednisone eq** | **0.9343** | **0.90 to 0.97** | **0.0006** |
| **Age** | 0.9970 | 0.98 to 1.01 | 0.7017 |
| **Sex (Male vs female)** | 0.9141 | 0.59 to 1.44 | 0.6945 |
| **Erosive RA (not-erosive vs erosive)** | 1.194 | 0.81 to 1.77 | 0.3751 |
| **Therapeutic line (≥2^nd^ vs 1^st^)** | 0.5532 | 0.30 to 1.02 | 0.0539 |
| **Antibodies (seropositive vs seronegative)** | 0.9788 | 0.66 to 1.45 | 0.9139 |

*Multiple binary logistic regression. ANOVA<0.0001; AUC: 0.64, 95% CI 0.58 to 0.69. Lower ORs correspond to lower chance of discontinuation.*

**Table s2.** Missing variables in the dataset

| **Variable** | **All cohort (n=716)** |
| --- | --- |
| **Age, n missing, % missing** | 0, 0% |
| **Sex, n missing, % missing** | 0, 0% |
| **Seropositive, n missing, % missing** | 12, 1.7% |
| **Erosive RA, n missing, % missing** | 0, 0% |
| **Smoking, n missing, % missing** | 17, 2.3% |
| **Monotherapy, n missing, % missing** | 5, 0.7% |
| **Taking glucocorticoids at baseline, n missing, % missing** | 0, 0% |
| **Glucocorticoid dose at baseline, mg/day, n missing, % missing** | 0, 0% |
| **DAS28-CRP at baseline, n missing, % missing** | 34, 4.7% |
